# Anti-fatigue Effects of Santé Premium Silver Perch Essence on Exhaustive Swimming Exercise Performance in Rats

**DOI:** 10.3389/fphys.2021.651972

**Published:** 2021-03-22

**Authors:** Chung-Yu Chen, Hei-Man Yuen, Chung-Chi Lin, Chi-Chieh Hsu, Jeffrey R. Bernard, Ling-Ni Chen, Yi-Hung Liao, Shiow-Chwen Tsai

**Affiliations:** ^1^Department of Exercise and Health Sciences, University of Taipei, Taipei City, Taiwan; ^2^Institute of Sports Sciences, University of Taipei, Taipei City, Taiwan; ^3^Healthcare and Service Center, Taipei Veterans General Hospital, Taipei City, Taiwan; ^4^Department of Exercise and Health Science, National Taipei University of Nursing and Health Sciences, Taipei City, Taiwan; ^5^Department of Aquatic Sports, University of Taipei, Taipei City, Taiwan; ^6^Department of Kinesiology, California State University-Stanislaus, Turlock, CA, United States; ^7^Anyong Biotechnology, Inc., Kaohsiung City, Taiwan

**Keywords:** exhaustive exercise, fish essence, myoglobin, liver glycogen, amino acids

## Abstract

**Aim:** Fish soup is a traditional Chinese food usually offered as a healthy supplement to elders, pregnant women and persons who just had surgery. Silver perch (Santé premium silver perch essence, SPSPE) extract contains various quality proteins, collagen, minerals, trace elements, and branch chain amino acids (BCAA) that could help individuals recover from exhaustion and control body weight. However, there are very limited studies exploring the effects of fish extracts on exercise performance and fatigue, and relevant physiological mechanisms. Therefore, the purpose of this study was to investigate the effects of chronic SPSPE administration on exhaustive exercise performance.

**Method:** Male Wistar rats weighing around 250 g were divided into 4 groups: Control, 1X SPSPE (6.2 ml/kg), 2X SPSPE (12.4 ml/kg) and 5X SPSPE (31.0 ml/kg). Rats were administrated SPSPE by oral gavage feeding every day for 33 days. Their body weight were measured every week. Before and after the exhaustive swimming test, the blood was collected for circulating lactate, glucose, ammonia, hormones, and myoglobin analysis. Rats were sacrificed after performing an exhaustive swimming exercise test. The liver tissues were collected for glycogen content and H&E staining.

**Results:** After the administration of 1X and 5X SPSPE, swimming fatigue was significantly delayed (*p* = 0.024). There was no difference in the hormone plasma level between the control and SPSPE groups. The induction of plasma corticosterone and TBARS by exhaustive swimming exercise could be decreased by SPSPE administration. The increased plasma myoglobin concentration from exhaustive swimming exercise was weakened by SPSPE supplementation. The higher glycogen sparing contained in liver tissue was observed in SPSPE-treated groups (*p* < 0.05).

**Conclusion:** SPSPE could efficiently delay swimming fatigue through sparing of liver glycogen and attenuation of plasma TBARS, myoglobin induction by exhaustive exercise. Our findings provide a scientific-based fundamental information and better understanding for developing a fish extract-based anti-fatigue supplement.

## Introduction

Exercise-induced fatigue is mainly associated with excessive exercise leading to fatigue and/or discomfort (Carroll et al., 2017). In highly intense exercise training or competitions, people receive energy regulated by the endocrinological and nervous systems, and the latter mainly controls muscle contraction. Carbohydrates are the main source of energy for sustaining endurance exercise (Gonzalez et al., 2016). The use of such energy is affected by exercise intensity, exercise duration, and nutrient status (Betts et al., 2008; Cermak and van Loon, 2013; Gonzalez et al., 2016). In humans, the main source of carbohydrates for muscles comes from the glycogen stored in muscle and liver during exhaustive exercise. The glycogen storage is closely related to fatigue induced by endurance exercise (Wei et al., 2019). The role of endocrine system plays during exercise metabolism is well-established, however, we are still learning about the exercise-induced changes to the endocrine system itself that are associated with exercise adaptation and fatigue recovery (Kraemer and Ratamess, 2005). For example, tissue damage caused by excessive exercise increases oxidative stress. When the body exhibits insufficient anti-oxidative capacity, it might not be able to counter the cytotoxic effect induced by the presence of excess free radicals (Droge, 2002). This situation begs the question of whether the endocrine system can be enhanced via the intake of specific nutrients to alleviate the fatigue caused by excessive exercise, thereby improving exercise performance.

In Eastern Asian countries, traditional foods such as broth made from pig bones, meat, or fish are used to accelerate the fatigue recovery of patients after surgery, in frail individuals, older adults, pregnant women, and breastfeeding mothers. Of these ingredients, fish flesh is composed of abundant unsaturated fatty acids, and its fat content is half that of pork. However, fish rots easily and generates substances that are harmful to the body. Therefore, an effective strategy that converts fish into easily preservable products such as fish protein hydrolysate and fish essence is necessary. Compared with whey protein hydrolysate, fish protein hydrolysate of equivalent weight exhibits higher total antioxidant capacity (Oliveira et al., 2020). Research on supplements created using fish extracts or essence has indicated that the ability of fish extracts to effectively enhance exercise performance and muscular strength is related to the presence of rich antioxidants (e.g., anserine and imidazole dipeptide carnosine), which reduce oxidative stress (Kikuchi et al., 2004).

Santé premium silver perch essence (SPSPE) is a supplement that is rich in protein and amino acids and is mainly constituted of branched-chained amino acids (BCAAs), a crucial nutrient for tissue synthesis, energy supply, and health maintenance (Li et al., 2009). Such amino acids also facilitate collagen repair and cell growth and affect exercise performance (Moore et al., 2005). Besides, a meta-analysis indicated that taking a BCAA-based supplement daily (>200 mg/kg of weight/day) for 10 days in a row can effectively alleviate mild and moderate muscular damage induced by exercise; this effect is particularly notable when the supplement is taken before high-intensity training (Fouré and Bendahan, 2017). Another recent study also reported that taking BCAA-based supplements 1 h before incremental treadmill exercise delays the occurrence of exhaustion (AbuMoh'd et al., 2020). However, few studies have explored the effects of fish extracts on exercise performance and fatigue, and relevant physiological mechanisms have yet to be fully clarified. Therefore, this study explores the effect of long-term perch essence supplementation on exercise performance and fatigue recovery in rats. The adequate supplement dose is also determined. In addition, we analyzed the physiological and biochemical mechanisms that potentially affect the benefits brought by perch essence.

## Materials and Methods

### Materials

The perch extract (essence) used in this study was produced by Yilan Anyong Lohas Co., Ltd. (Yilan, Taiwan). The perch essence was stored in a refrigerator at 4°C to ensure the quality of storage. Herbiotek Co., Ltd. was commissioned to analyze the nutrient content of the perch essence. The analysis results are listed in Table 1. The essence was determined to contain 90 mg/mL protein and a total BCAA content of 7.14 mg/mL. The recommended daily perch essence intake for an adult weighing 60 kg is 60 mL (1 mL/kg of weight). According to the human equivalent dose proposed by the US Food and Drug Administration, the rat equivalent dose is ~6.2 times that of humans. A conversion coefficient of 6.2 was therefore employed in this study to determine the perch essence dose for rats 6.2 mL/kg in weight (Wojcikowski and GobeShin, 2014).

**Table 1:** Nutrition facts, hydrolyzed amino acid profiles and total branched-chain amino acids of fish essence.

**Nutrition Facts**	**Content (/100 ml SPSPE)**
Protein	9 g
Fat	0.033 g
Saturated fat	0.017 g
Trans fat	0 g
Cholesterol	0 mg
Carbohydrate	0 g
Sodium	98.33 mg
Total calories	36.1 Kcal
**Hydrolyzed amino acid profiles**	**mg/100 ml SPSPE**
Alanine	837.56
Phenylalanine	222.33
Cysteine	141.67
Aspartic acid	561.27
Glutamic acid	1,077.86
Glycine	1,539.95
Histidine	116.58
Leucine	294.97
Isoleucine	131.72
Lysine	339.63
Methionine	173.03
Proline	1,121.0
Arginine	612.22
Serine	243.06
Threonine	293.82
Valine	201.93
Tryptophan	11.09
Tyrosine	88.31
**Total BCAA**	**mg/100 ml SPSPE**
Valine, leucine and isoleucine	628.62

### Animal Care and Experimental Design

Six weeks-old Wistar rats (BioLASCO, Yilan, Taiwan) were given 2 weeks to adapt to the laboratory environment and then being assigned according to their weights into four *groups*, namely the control group (*n* = 9), low dose group (1X dose, or 6.2 mL/kg of weight; *n* = 9), medium-dose group (2X dose, or 12.4 mL/kg of weight; *n* = 9), and high dose group (5X dose, or 31.0 mL/kg of weight; *n* = 9). During this experiment, the rat drinking water was replaced daily. Rats were given access to food and water *ad libitum*. The room temperature was controlled between 21 and 23°C and the humidity between 50 and 60%. The lights were switched on or off every 12 h to simulate a 12-h day-night cycle. The cage bedding was replaced twice a week, and the cages were cleaned weekly. All animal experiments performed in this study were approved by the Institutional Animal Care and Use Committee of the University of Taipei (UT108004).

**Figure 1:**
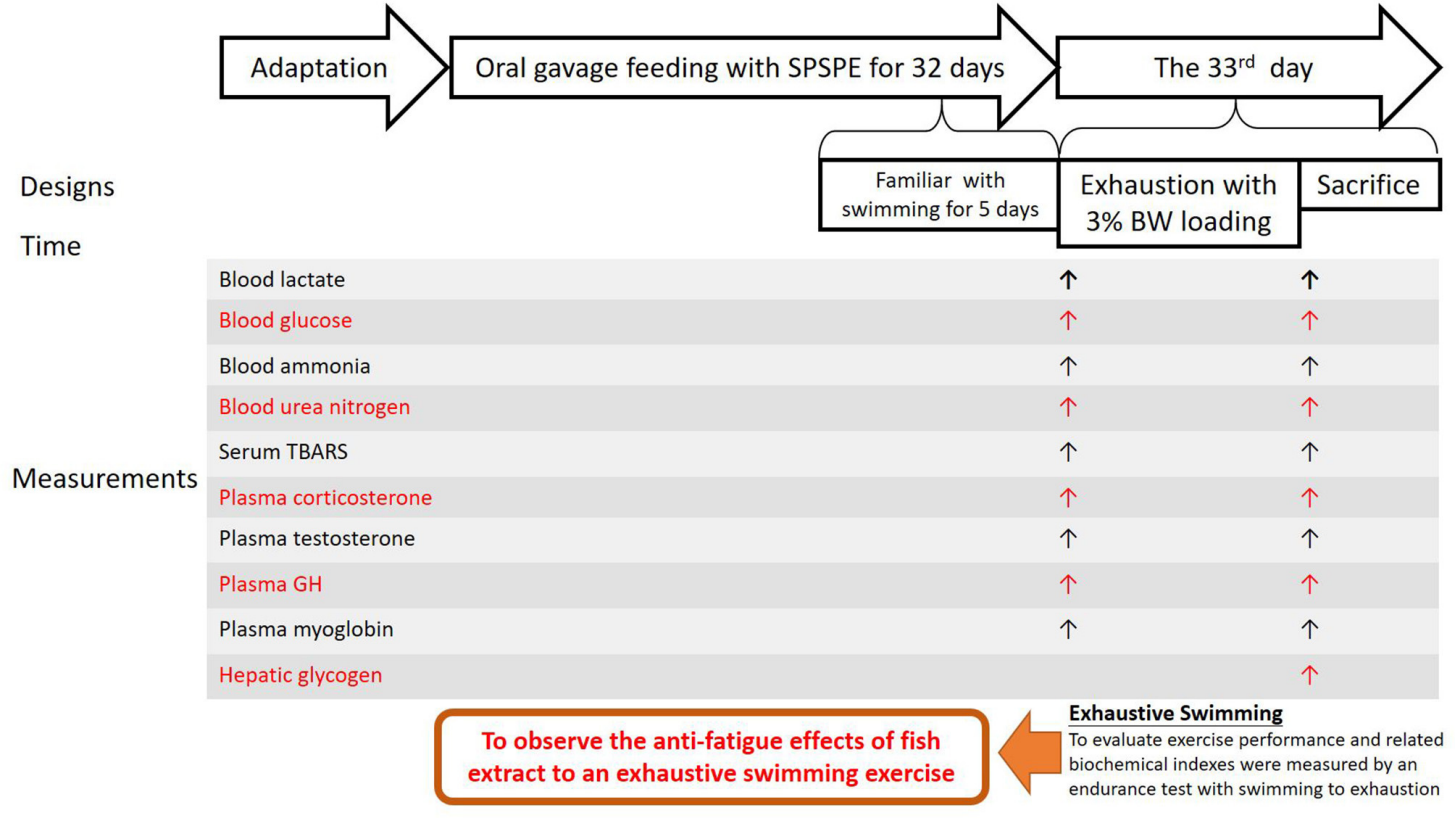
Procedure and measurement of experimental design.

As shown in Figure 1, after the rats were relocated to their respective cages (two rats per cage), they were given another week to become familiar with the cage environment and oral gavaging operation before perch essence supplementation was begun. Before all the experiments, the animals were familiarized with oral gavaging operation for at least 1 week to eliminate the possible stress interference for this procedure. The initial body weights of each group at the time of perch extract administration are shown as follow: the control group (control: 260.67 ± 1.76 g), low dose group (1X dose: 261.89 ± 2.66 g), medium-dose group (2X dose: 265.50 ± 3.92 g), and high dose group (5X dose: 270.44 ± 3.96 g). And all processes are operated by well-trained laboratory colleagues, and all animals were received the familiarization gavaging procedure for 7 days to eliminate the related stress response during acclimation period. After assigning animal treatment groups, the same gavaging operation was performed regardless of taking either placebo or treatment supplement to ensure the consistency of the experiment. The control group also received identical sham gavage as other animals with supplementation substance. We also followed the recommendation of ~10 ml/kg for each gavaging supplement in rats. The total daily amount was delivered by separating to three times, and the individual providing doses were ranged between 2.1 ml/kg (1X) and 10.3 ml/kg (5X) for each gavaging operation. Although the amount was very close to the upper limit of the recommend gavaging amount, we still tried best to minimize the possible stress during our procedure. The experimental period spanned 33 days, during which the rats received a standard chow diet (5001, PMI Nutrition International, MI, USA).

In this study, the main purpose was to observe the anti-fatigue effects of fish extract to an exhaustive swimming exercise. After oral gavage feeding for 32 days, the rats were fasted for 12 h and underwent an exhaustive swimming exercise endurance test in which they were loaded with a weight equivalent to 3% of their body weight. On the day of the test, the rats began the test 60 min after receiving oral gavaging. Before and after the exhaustive swimming test, the venous blood samples were collected from the tail to measure the following circulating parameters: lactate, glucose, ammonia, thiobarbituric acid reactive substances (TBARS), corticosterone, testosterone, growth hormone, myoglobin, and blood urea nitrogen (BUN) concentrations. The rats were sacrificed immediately after the test to collect liver samples to analyze the glycogen content and perform histological staining.

### Exhaustive Swimming Exercise Test

Herein we used the overnight fasting that has been commonly utilized in metabolic and exercise-related research to ensure that there are no interference factors (Wu et al., 2013). In this study, we thus carried out 12-h fasting to minimize the possible impacts of uncertain time food intake on metabolic changes and subsequent exercise performance, and the rats received their final corresponding doses of oral SPSPE gavaging at 60 min in prior to swimming test. One week before the formal test the rats swam for 10 min per day for 5 days in a row without any weight load to become accustomed to swimming. The pool was 53 cm in diameter and 60 cm in-depth, and the water temperature was set at 27 ± 1°C as the formal swimming exercise test. After 33 days of the perch essence intervention, the rats underwent the exhaustive swimming exercise endurance test, during which a load equivalent to 3% of their weight was tied to their tails. The water temperature was 27 ± 1°C. We recorded the time at which each rat began to swim and the time they could not sustain swimming and be completely exhausted. The following standards were used to determine whether a rat was completely exhausted: the rat could not (1) continue the swimming movement (2) or reach the water surface within 8 s after becoming submerged. After the test, tail venous blood samples were collected before the rats were sacrificed immediately.

### Biochemical Profiles and Plasma Hormone Content

The collected blood samples were tested using a blood glucose meter (GE100, Taiwan) and a blood lactate meter (The EDGE, Taiwan). Blood ammonia and blood urea nitrogen were determined using colorimetric analysis. The blood samples were also centrifuged at 4°C to acquire plasma samples. The enzyme-linked immunosorbent assay (ELISA) was used to determine the plasma corticosterone (Immuno-Biological Laboratories, Inc., NE, USA), testosterone (Cayman, MI, USA), growth hormone (Société de Pharmacologie et d'Immunologie-BIO, France), and myoglobin concentrations (Immunology Consultants Laboratory, Inc., OR, USA). Reaction agents were loaded into all samples according to the respective instructions from the suppliers, and an ELISA analyzer (TECAN Infinite R200 PRO, Switzerland) was used to determine the optical density (OD). The obtained values underwent standard regression plot standard curves, which were used to determine the sample concentrations through interpolation. The TBARS concentration was measured using a chemical kit (Cayman, MI, USA).

### Tissue Glycogen Determination

The largest liver lobe and white gastrocnemius muscle from each rat was also preserved at −80°C to perform subsequent glycogen content analysis using a hydrolysis enzyme decomposition kit (Glycogen Colorimetric/Fluorometric Assay Kit; BioVision Incorporation, CA, USA). Specifically, 50 mg of a liver lobe sample and 50 mg of white gastrocnemius muscle sample were ground on an icy surface and mixed with 500 μL of cold water. The mixture was then boiled in a boiling water bath for 10 min to destroy the enzymes in the tissue sample. The mixture was then centrifuged to acquire the supernatant, which was mixed with reagents, reacted in the dark for 30 min, and measured at 570 nm. The concentration was determined using regression and interpolation.

### Histological Tissue Staining

We collected liver tissue samples from the outer areas of the left liver lobe, fixed the samples in 10% formalin, and coated in paraffin wax. The coated samples were sliced into 4-μm-thick sections to perform hematoxylin and eosin staining. The sections were examined by veterinarians using optical microscopes with charge-coupled cameras (Olympus BX-51, Tokyo, Japan).

### Statistical Analysis

All experimental data were used SPSS 22.0 software (SPSS, Chicago, IL, USA) to analyze and presented as mean ± standard errors mean. The Levene test was used to verify the hypothesis of homogeneity between groups, and the Kruskal-Wallis test was used to examine the mean between groups that did not conform to the normative distribution. Because the samples did not conform to the normative distribution, we used a more conservative statistical analysis for multiple tests (i.e., Kruksal-Walis with Bonferroni correction) to compare the means between groups. The significance level was set as *p* < 0.05.

## Results

### The Body Weight

**Figure 2:**
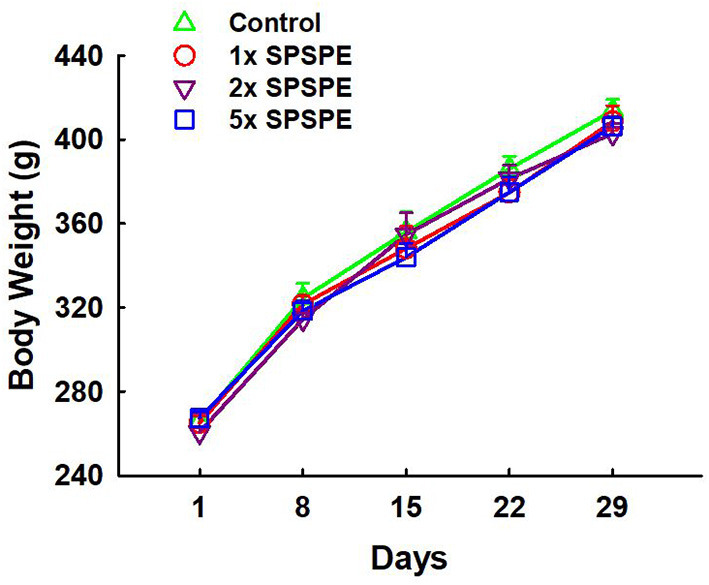
The effects of SPSPE treatment on body weight. Each value represents Mean ± SEM, *n* = 9.

The changes in body weight in each group were measured weekly and shown in Figure 2. The body weight in each group increased from 250 to 400 g after treatments for 29 days. There was no significant difference among the control and SPSPE treated groups.

### The SPSPE Supplementation Effects on Exercise Performance

**Figure 3:**
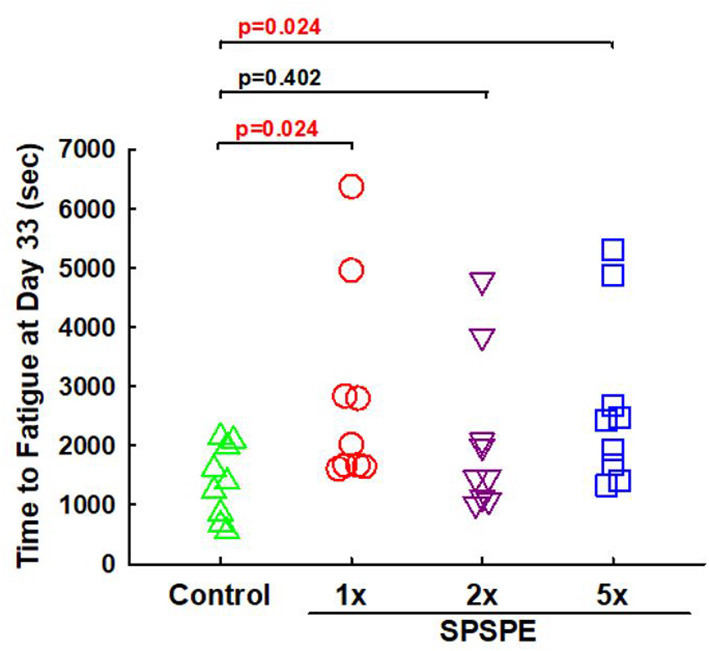
Effect of SPSPE treatment on the results of swimming exhaustive test in rats. After treatment for 32 days, the rats were fasting for 12 h. The next day, the rats were administrated with different doses of SPSPE and stayed in their cages for 60 min, and then the rats performed a swimming exhaustive test.

On day 33, the rats received their final corresponding doses of oral SPSPE gavaging after an overnight fasting. After 60 min the rats began the swimming endurance test as an indicator of endurance performance. In the SPSPE treated groups the endurance performance was significantly higher than that of the control group (Figure 3). In the control group the longest swimming time was 2,149 s. The mean values were 1,394 s and the median value was 1,390 s. In the 1X SPSPE group the swimming time ranged from 1,610 to 6,381 s. The median value was 2,020 s which is significantly higher than that of the control group (*p* = 0.024). In the 2X SPSPE group the swimming time was distributed between 1,014 and 3,845 s. The median value was 1,467 s with no significant difference compared to the control group (*p* = 0.402). In the 5X SPSPE group the swimming time ranged from 1,331 to 5,314 s. The median value was 2,429 s which is significantly higher than the control group (*p* = 0.024).

### SPSPE Effects on Circulating Biomakers to the Exhaustive Swimming Exercise Test

**Figure 4:**
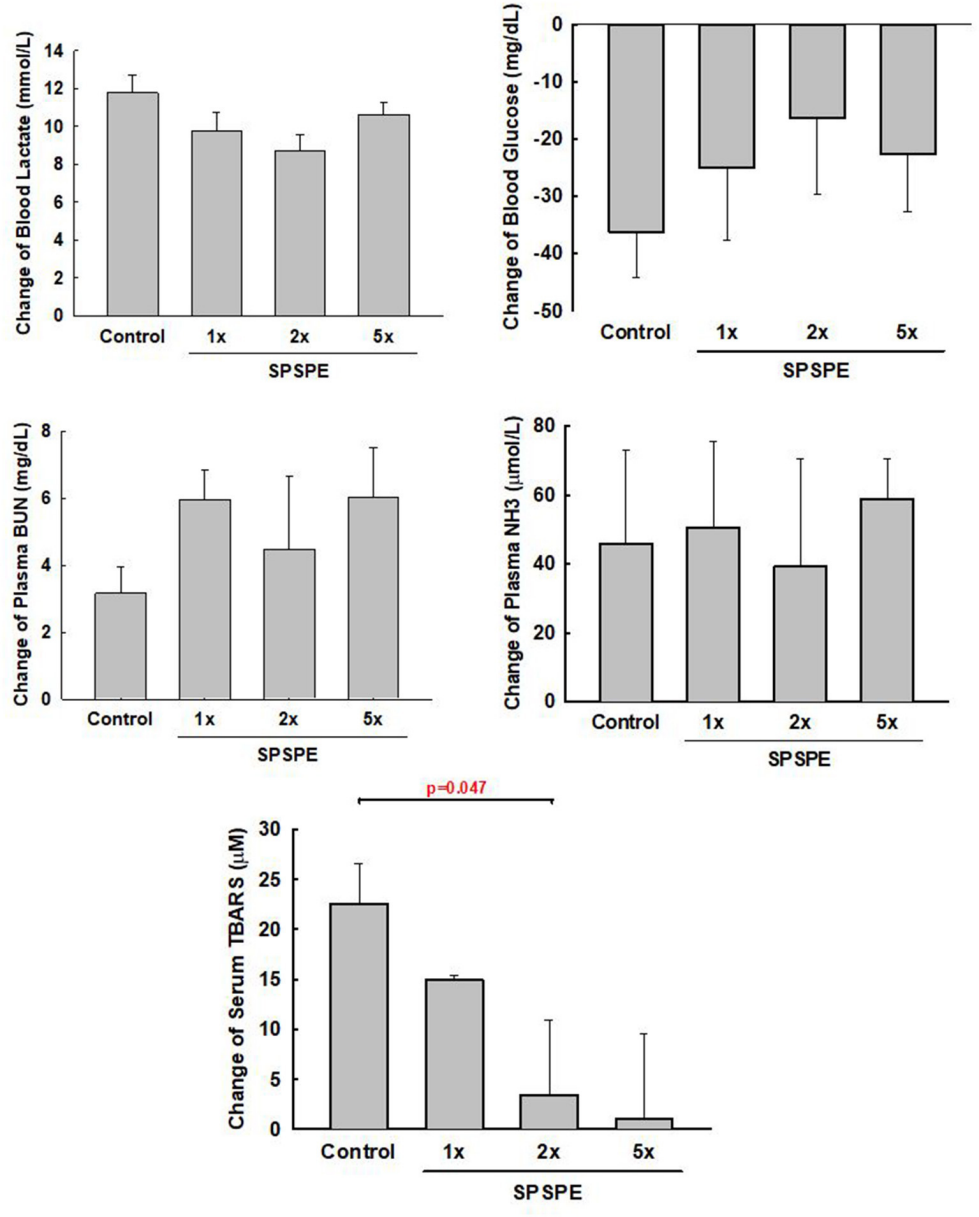
The rats were administrated with different doses of SPSPE for 33 days. The change of circulating lactate, glucose, BUN, NH3, and TBARS after exhaustive swimming exercise test is shown each value represents Mean ± SEM, *n* = 9.

After the exhaustive swimming exercise test, the changes in serum TBARS after the exhaustive swimming test (as compared to the serum level of TBARS before the exhaustive swimming testing) are shown in Figure 4, although all other measured biomarkers (i.e., BUN, ammonia, blood glucose, and lactate) did not exhibit any statistical differences among treatments. The elevated serum TBARS decreased in the SPSPE treated groups, and a significant difference could be observed in the 2X SPSPE group (*p* = 0.047).

### SPSPE Effects on the Exhaustive Swimming Exercise Test Induced Plasma Level of Hormones

**Figure 5:**
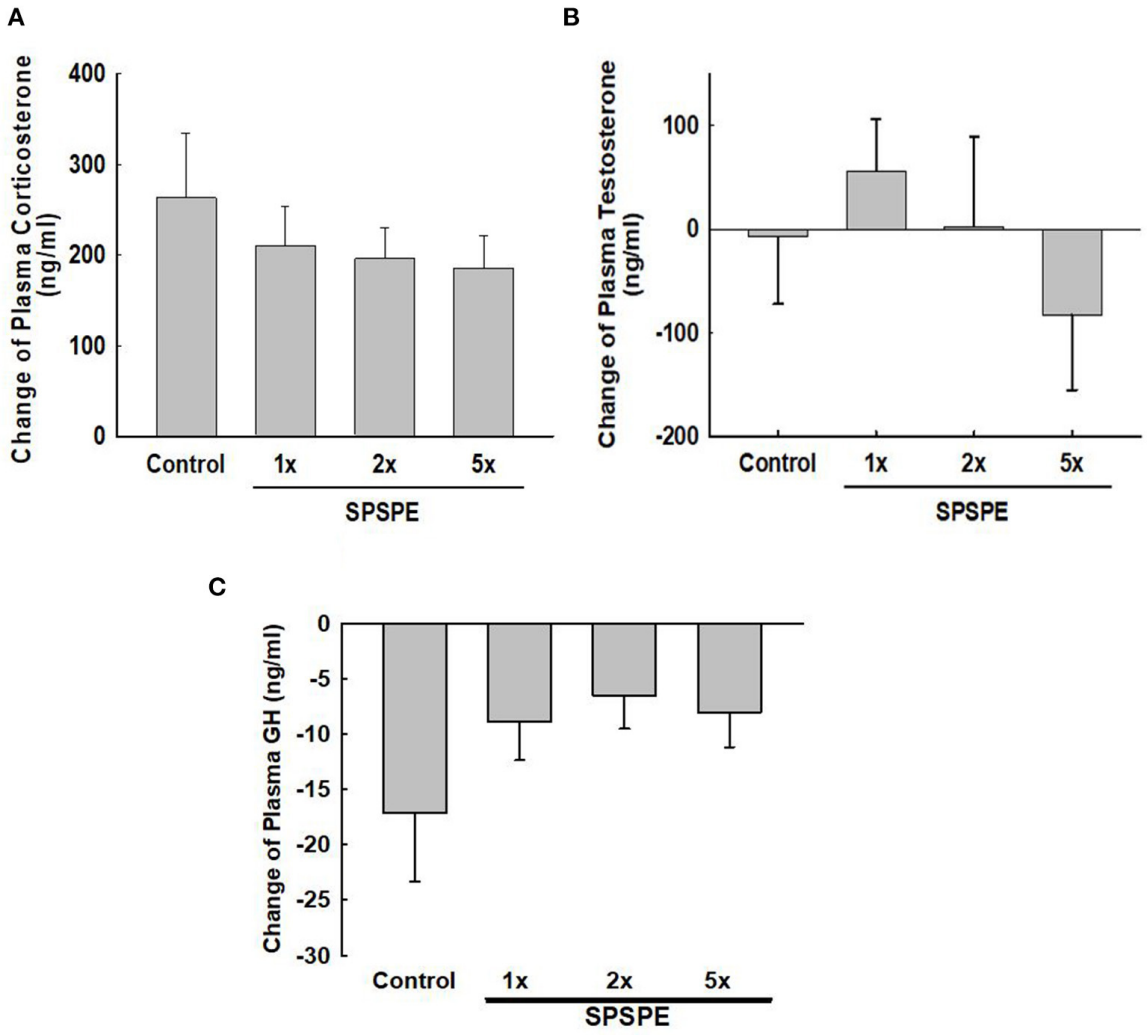
The effects of SPSPE treatment on the plasma level of corticosterone **(A)**, testosterone **(B)**, and growth hormone **(C)** in rats after exhaustive swimming exercise test, each value represents Mean ± SEM, *n* = 9.

Before and after the exhaustive swimming test, blood was collected and corticosterone, testosterone, and growth hormone were tested in serum or plasma by enzyme immunoassay. The changes in corticosterone concentration induced by exhaustive exercise were no significant difference among groups (Figure 5A). Likewise, the changes in testosterone concentration after swimming exercise also exhibited no significant differences among groups (Figure 5B). Additionally, the SPSPE treatment did not alter the changes in plasma growth hormone response after exhaustive swimming exercise (Figure 5C).

### SPSPE Effects on Circulating Myoglobin Levels in Response to the Exhaustive Swimming Exercise Test

**Figure 6:**
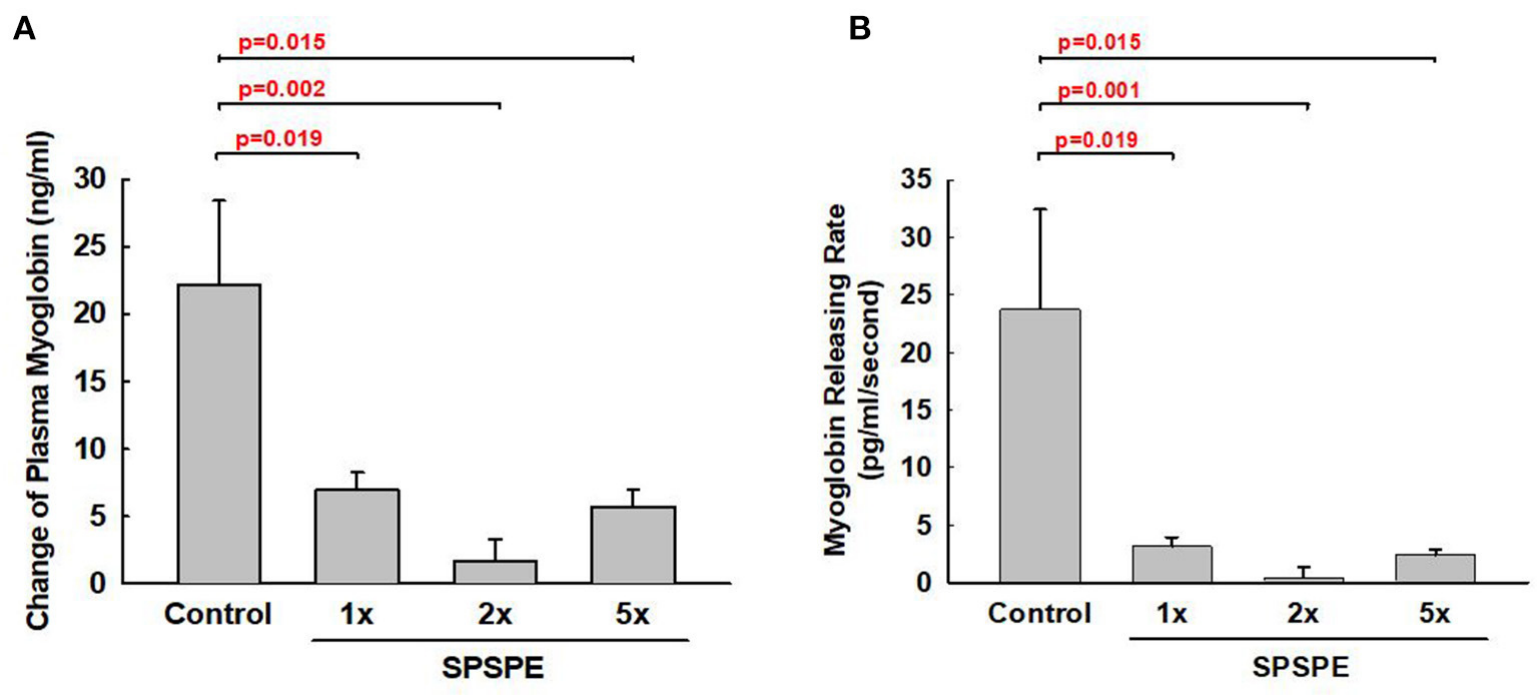
The effects of SPSPE treatment for 33 days on the plasma level of myoglobin **(A)** and myoglobin releasing rate **(B)** after an exhaustive swimming exercise test, each value represents Mean ± SEM, *n* = 9.

Exhaustive exercise could cause muscular damage and release myoglobin into the blood. The SPSPE treatment significantly attenuated the exhaustive exercise-induced changes in plasma myoglobin level (Figure 6A). Furthermore, we calculated the myoglobin releasing rate (pg/ml/second) and found that the myoglobin release rate in SPSPE supplemented groups were significantly lower than that in the control group (Figure 6B).

### SPSPE Supplementation Effect on Liver and Muscle Tissue Glycogen Levels

**Figure 7:**
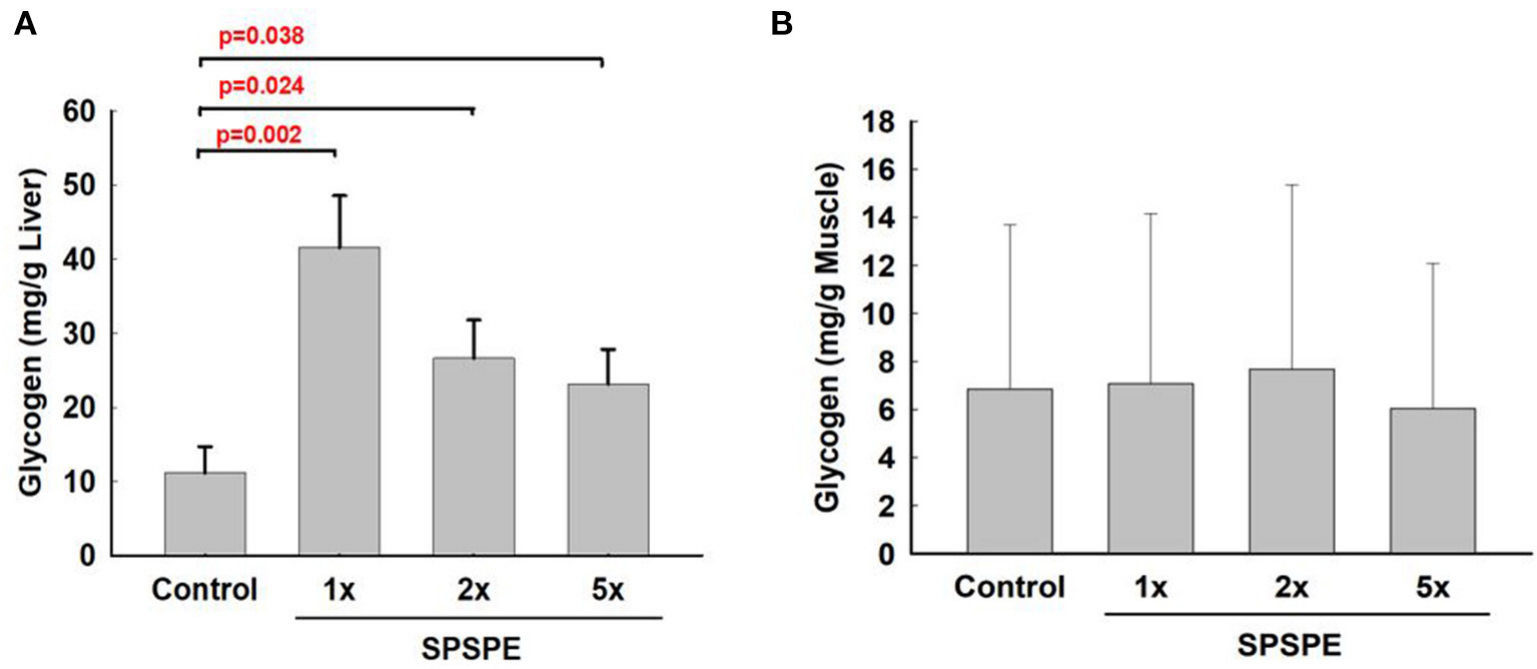
The influence on glycogen content in liver tissue **(A)** and white gastrocnemius muscle **(B)** after treatment of SPSPE for 33 days, each value represents Mean ± SEM, *n* = 9.

After exhaustive exercise, the rats were sacrificed and the liver and white gastrocnemius muscle were extracted for the glycogen measurements. After oral gavaging SPSPE for 33 days, glycogen sparing in the liver was significantly increased compared with the control group (Figure 7A). However, the SPSPE supplementation did not exhibit any beneficial effects on sparing muscle glycogen after exhaustive swimming exercise (Figure 7B).

### Correlation Analyses

For the correlation analyses, we pooled the data from all treatment groups to further analyze the correlations among certain anti-fatigue related parameters. The results showed that only the increase of exhaustive time was negatively correlated with the releasing rate of myoglobin per unit of time in response to the exhaustive swimming exercise (*r* = −0.317, *p* = 0.023). The correlation between exhaustive time and change of lactate concentration was approaching significant (*r* = −0.276, *p* = 0.052). There were no significant correlation between swim performance and TBARS (*r* = −0.094, *p* = 0.283), growth hormone (*r* = −0.129, *p* = 0.214), hepatic glycogen content (*r* = 0.043, *p* = 0.398).

### Assessments of Histopathology

**Figure 8:**
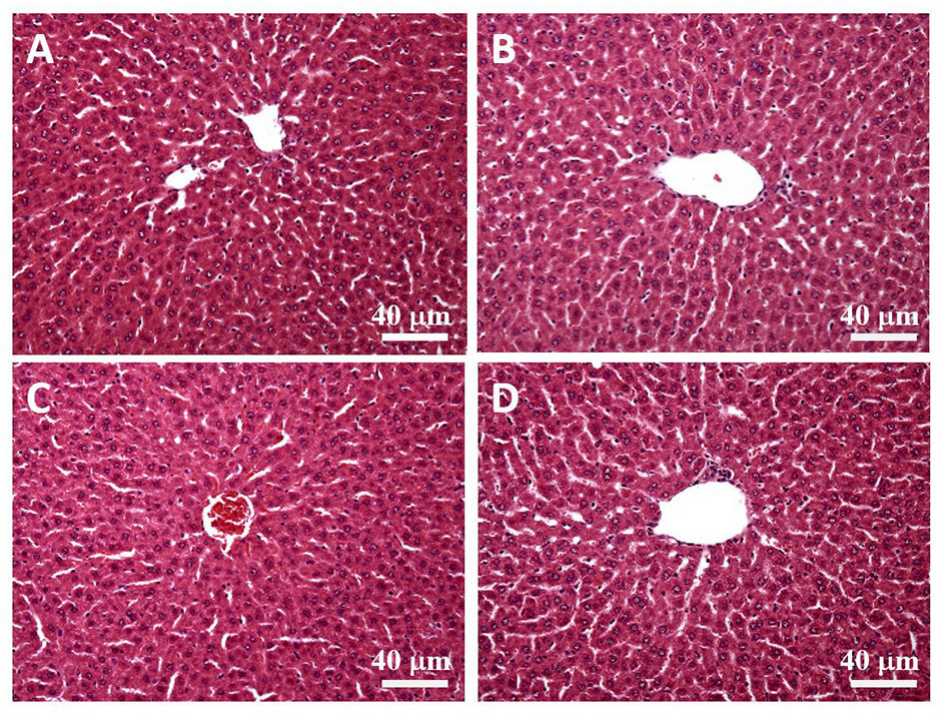
The liver morphology in control **(A)** and SPSPE treated groups (**B**: 1X, **C**: 2X, **D**: 5X). The H & E stain was performed and the slices were observed under 200x magnification.

H&E stain was performed after the liver tissue was sliced. By observing the liver plates near the central vein (eyepiece 10 times, objective lens 20 times), we found that the liver plates arranged in a radial pattern in the control and SPSPE treated groups. No pathological changes were shown after SPSPE administration (Figure 8).

## Discussion

The results revealed that after the rats were fed perch essence continually for 33 days, their swimming endurance performance was significantly improved. The rats effectively maintained glycogen content following the swimming exercise. Plasma TBARS formation decreased with increasing perch essence intake, which also significantly reduced myoglobin formation per unit time of exhaustive exercise. The aforementioned results indicate that the perch essence effectively enhances exercise performance through several possible mechanisms, such as liver glycogen sparing, muscle damage reduction, and oxidative stress acceleration.

Carbohydrates and fats are the main sources of energy for humans when they perform endurance exercise (Gonzalez et al., 2016), and the similar responses are also well-recognized in rat (Baldwin et al., 1975; Fitts et al., 1975; Clark and Conlee, 1979; Dohm et al., 1983). The utilization ratio of these two substances depends on exercise intensity, exercise duration, and nutrient status (Betts et al., 2008; Cermak and van Loon, 2013; Gonzalez et al., 2016). The body usually stores carbohydrates as glycogen in the liver and muscles. Research has indicated that during moderate- and high-intensity endurance exercise that spans an extensive period, glycogen in the liver and muscles is the main source of energy. Therefore, glycogen storage in the body and fatigue during endurance exercise are closely related (Wei et al., 2019). Compared with the control group, the rats receiving 1X, 2X, and 5X doses of the perch essence exhibited significantly higher glycogen concentrations after the exhaustive swimming exercise. This hints at the possibility that perch essence facilitates effective energy utilization (i.e., comes from lipids and proteins) during exercise and induces a glycogen-sparing effect, which delays the occurrence of fatigue. On the other hand, we did not determine the initial glycogen levels for 1X, 2X, and 5X groups, thus we still cannot rule out the possibility whether the chronic administration would alter the initial hepatic glycogen levels.

During intense exercise or long-term training, biochemical variables might change considerably. Acute endurance exercise (e.g., intense swimming exercise) might substantially increase blood indicators, such as lactate dehydrogenase, aspartate aminotransferase, creatine kinase, myoglobin, and bilirubin (Wu et al., 2013). These indicators are associated with postexercise heart and muscle damage and often increase continually after exercise (Nie et al., 2011). Different experimental models have been adopted to identify multiple nutrient supplements that can effectively reduce these indicators. However, few studies have explored whether perch essence can exert long-term effects on these indicators when performing exercise challenges. Healthy participants were recruited to receive eccentric contraction-induced muscle damage. Their muscle strength deterioration and lactate dehydrogenase levels were reduced after consuming whey protein (Cooke et al., 2010). In our study, the exhaustive swimming exercise significantly increased the myoglobin level, a blood indicator associated with muscle damage (Figure 6A). This finding is consistent with those of relevant studies (Sabriá et al., 1983). Our study provides further evidence that long-term intake of perch essence can effectively reduce myoglobin formation per unit time of exhaustive exercise. The drastic increase in plasma myoglobin content might indicate oxidative damage, physical cellular damage, or other disease symptoms, which could cause myoglobin leakage from damaged muscle cells (Kiessling et al., 1981; Sabriá et al., 1983; Driessen-Kletter et al., 1990). Another study verified that long-term soy protein intake is conducive to reducing muscle damage caused by exhaustive exercise (reducing serum creatinine phosphate kinase concentration) and enhancing exercise capacity recovery (Wei et al., 2019). Similarly, we also observed that perch essence significantly reduced myoglobin level related to muscle damage. This indicates that perch essence can protect skeletal muscles from damage induced by intense exercise. This beneficial effect might also contribute to the improved swimming performance caused by perch essence.

Tissue damage caused by exhaustive exercise might lead to increased oxidative stress. When the endogenous anti-oxidative system of the body cannot react adequately, it might not be able to offset the cytotoxic effect caused by excess free radicals (Droge, 2002). The oxygen consumption of muscle tissues during exercise increases the formation of free radicals. The reduction of free radical removal ability might induce oxidative damage in cellular biomolecules, including those in cell membranes, cytoplasm, and DNA (Aoi et al., 2004; Bessa et al., 2016; Jamurtas, 2018). TBARS is metabolic byproducts of phospholipid peroxidation and is often used as a biological indicator to assess oxidative damage in organisms (Yada et al., 2020). Besides, several studies have observed significant increases in the plasma lipid peroxidation level after exhaustive aerobic exercise (Waring et al., 2003; Watson et al., 2005). In this study, the adopted exhaustive swimming exercise significantly increased oxidative stress in rats. Compared with other groups, the control group exhibited a greater increase in the plasma lipid peroxidation level after exercise (Figure 4). A 2-fold increase in the perch essence dose significantly reduced this increase in TBARS after the exhaustive swimming exercise (*p* = 0.047, Figure 4). Other studies verified that excess free radical or reactive oxygen species produced from intense exercise exacerbate muscle damage and fatigue affect skeletal muscle contraction (Alessio et al., 2000; Mastaloudis et al., 2001; Powers et al., 2004). Together with previous studies (Alessio et al., 2000; Mastaloudis et al., 2001; Powers et al., 2004) and our present findings, long-term perch essence intake is recommended before performing an intense high-volume exercise to reduce lipid peroxidation induced by the exercise.

Highly intense or long exercise periods affect endocrine system regulation. A study concluded that the changes in the endocrine system and adaptation responses following exercise are related to fatigue recovery (Kraemer and Ratamess, 2005). Previous studies have indicated that increasing the secretion of anabolic hormones following intense exercise can accelerate recovery from fatigue (Kraemer and Ratamess, 2005; Peake et al., 2005). Growth hormone and testosterone are anabolic hormones secreted by the human body and are conducive to muscle strength development, muscle growth, protein synthesis, and damaged tissue repair. Studies have discovered that exercise is a crucial factor in increasing the secretion of growth hormone and testosterone. After exercise, the secretion of these two hormones is increased, which in turn regulates the synthesis of muscle proteins, accelerates the recovery of muscle strength, and facilitates tissue repair (Ferrando et al., 2002; Saugy et al., 2006). In this study, we observed that the secretion of testosterone (Figure 5B) and growth hormone (Figure 5C) was not affected by perch essence intake, indicating that the perch essence-induced endurance enhancement did not mediated through further increasing exercise-induced anabolic hormones.

Herein we observed that the glycogen sparing effects of SPSPE supplementation were only existing in the liver tissue instead of skeletal muscle after exhaustive swimming exercise (Figures 7A,B). The reason we measured the white muscle fiber was considering that the white fibers are recruited when strenuous work is performed or when red fibers become fatigued, indicating that the glycogen levels in white fiber would better represent the energy substrate utilization and sparing at the end of exhaustive exercise (Baldwin et al., 1975; Fitts et al., 1975; Clark and Conlee, 1979; Dohm et al., 1983). According to the present findings, we speculate that the SPSPE spares liver glycogen content, thereby possibly better sustaining glucose availability during prolonged exercise. Subsequently, animals with SPSPE treatment had better sparing of liver glycogen which may indicate that fat oxidation is mainly during exercise rather than anaerobic metabolism of carbohydrates. But another possibility may also be due to the supplement of SPSPE before the exhaustive exercise. Since branched-chain amino acids are the main components in the SPSPE, it may help reduce the central fatigue state of experimental animals (AbuMoh'd et al., 2020). Previous reports have shown that there is no decline in the submaximal or maximal cycling performance after caloric restriction for 3 weeks combined with overnight fasting (Ferguson et al., 2009), suggesting that overnight fasting before our swimming test should not affect outcome measurements of this study. Furthermore, our study is mainly to explore the impact of long-term administration of fish extract on exercise performance and anti-fatigue ability. Therefore, we believed that the single overnight fasting should be required to eliminate possible confounding factors without affecting chronic treatment effects. Also, our findings that the changes in performance and related biomarkers should be mainly reflected by our treatment supplement and dosage.

When using specially processed food (e.g., nutrient supplements) as a health food, food safety is the most crucial consideration. Currently, no study has explored the safety of perch essence, which is primarily composed of protein-based amino acids. Nutrient supplements with protein or amino acids have been prevalently taken by athletes to improve their adaptation to exercise intensity. Moreover, studies have reported that no undesirable effect was observed when consuming as much as 3 g·kg^−1^·d^−1^ of protein (Dyer et al., 2008), and that food additive made of 2 g·kg^−1^ of protein hydrolysates do not increase the fatality rate (Anadón et al., 2013). In the present study, the 1X-5X doses given to the rats were equivalent to 0.56–2.70 g·kg^−1^·d^−1^ of protein, which did not exceed the safe dose range determined in previous research. Moreover, the liver tissue samples of rats in the 1X, 2X, and 5X dose groups did not exhibit any undesirable reactions (Figure 8). Therefore, the biochemical variables measured in this study can accurately reflect the physiological effect and fatigue alleviation caused by the perch essence.

Fish extract contains essential amino acids (EAAs) and 17–20% protein (Kietzmann et al., 1969; Ludorff and Meyer, 1973; Mol, 2005), and our fish extract analyses also exhibited a rich content of EAAs, especially a high percentage of BCAAs (714 mg/100 mL). In addition, fish muscle is also easily digestible due to its inherent connective tissue content (~2%) (Kietzmann et al., 1969; Ludorff and Meyer, 1973). Moreover, some fish tissues contain fat-soluble vitamins and numerous fish products are rich in water-soluble vitamins (Ludorff and Meyer, 1973). Since previous studies have previously shown that protein or amino acid supplementation can significantly reduce exercise-induced fatigue, suppress muscle soreness, and promote recovery from high-intensity exercise (Cooke et al., 2010; Fouré and Bendahan, 2017; Wei et al., 2019; AbuMoh'd et al., 2020); furthermore, certain fat- and water-soluble vitamins have been reported to have antioxidant effects and be capable of reducing the degree of exercise-induced muscle damage (Evans, 2000; Thompson et al., 2001; Taghiyar et al., 2013). Although our nutrient analyses did not perform vitamin analyses, we speculated that the SPSPE supplementation reduces exercise-induced fatigue and muscle protective effects possibly mediated through the above possible benefits of fish-containing nutrients. Future investigations are warranted to analyze the vitamin contents to better understand the possible potential benefits of fish extract.

There are still several limitations in this study. Here a total of nine animals in each group were included to test the dose-response of SPSPE on the anti-fatigue effects during exercise. Although many animal studies investigating nutrients supplementation also used comparable number of animals as our current investigation, it is warranted to conduct experiments with a larger sample size in the future. Moreover, according to the findings in our measured biomarkers, we were not able to provide the explanation for our observations that only 1X and 5X doses were efficient at improving time to exhaustion but not 2X dose. Still, we could not exclude the possible benefits of this BCAA-enriched SPSPE on reducing the central fatigue state (AbuMoh'd et al., 2020), thus the role of central fatigue in this observation is needed to investigate for better understanding about the possible underlying mechanisms. Furthermore, it has to be noted that we here used a more conservative statistical analysis to compare the mean among groups when performing multiple tests (i.e., Kruksal-Walis with a Bonferroni correction). Therefore, there might be some existing possible benefits of SPSPE administration that could be masked using such conservative statistical analysis. In the future, more empirical studies are warranted to translate the applications in general or athletic human populations.

## Conclusion

In summary, we examined the biochemical indicators related to fatigue and recovery in rats performing an exhaustive swimming exercise. Continual intake of perch essence for 33 days significantly extended their swimming duration. Moreover, perch essence supplementation effectively maintained their glycogen level after exhaustive exercise. According to our present results, the effective dose is 6.2–12.4 ml per kilogram of rat body weight/day, which is equivalent to 1–2 ml per kilogram of human body weight/day. Meanwhile, perch essence markedly reduced the formation of TBARS and myoglobin during exercise. Also, we observed that the increase in exhaustive time was negatively associated with the appearance rate of circulating myoglobin during the exhaustive swimming exercise. Furthermore, we performed pathological observation of the liver, and provided evidence regarding the safety of perch essence, verifying its feasibility as an alternative nutrient supplement for reducing exercise fatigue.

## Data Availability Statement

The raw data supporting the conclusions of this article will be made available by the authors, without undue reservation.

## Ethics Statement

The animal study was reviewed and approved by Institutional Animal Care and Use Committee of the University of Taipei.

## Author Contributions

C-YC, H-MY, Y-HL, and S-CT: conceptualization and writing—original draft preparation. C-CL, C-CH, and JB: methodology and data curation. C-YC, H-MY, and Y-HL: validation. S-CT: formal analysis and funding acquisition. C-YC, H-MY, C-CL, C-CH, and JB: investigation. S-CT and L-NC: resources. C-YC, Y-HL, and S-CT: writing—review and editing. Y-HL and S-CT: supervision. H-MY: project administration. All authors have read and agreed to the published version of the manuscript.

## Conflict of Interest

S-CT is a Professor at University of Taipei, Taipei City, Taiwan, and she received the research funding from Yilan Anyong Lohas Co., Ltd. (Yilan, Taiwan) (grant number: 108471). Researchers at University of Taipei are responsible for the entire animal study and report. The remaining authors declare that the research was conducted in the absence of any commercial or financial relationships that could be construed as a potential conflict of interest.
